# The Importance of Close Follow-Up in Patients with Early-Grade Diabetic Retinopathy: A Taiwan Population-Based Study Grading via Deep Learning Model

**DOI:** 10.3390/ijerph18189768

**Published:** 2021-09-16

**Authors:** Chia-Cheng Lee, Shi-Chue Hsing, Yu-Ting Lin, Chin Lin, Jiann-Torng Chen, Yi-Hao Chen, Wen-Hui Fang

**Affiliations:** 1 Planning and Management Office, Tri-Service General Hospital, National Defense Medical Center, Taipei 11490, Taiwan; clee112@ndmctsgh.edu.tw; 2 Division of Colorectal Surgery, Department of Surgery, Tri-Service General Hospital, National Defense Medical Center, Taipei 11490, Taiwan; 3 Department of Internal Medicine, Tri-Service General Hospital, National Defense Medical Center, Taipei 11490, Taiwan; lars0121@gmail.com; 4 Division of Cardiovascular Surgery, Department of Surgery, Tri-Service General Hospital, National Defense Medical Center, Taipei 11490, Taiwan; louie0511@hotmail.com; 5 Graduate Institute of Life Sciences, National Defense Medical Center, Taipei 11490, Taiwan; xup6fup0629@gmail.com; 6 School of Medicine, National Defense Medical Center, Taipei 11490, Taiwan; 7 School of Public Health, National Defense Medical Center, Taipei 11490, Taiwan; 8 Department of Ophthalmology, Tri-Service General Hospital, National Defense Medical Center, Taipei 11490, Taiwan; jt66chen@gmail.com (J.-T.C.); doc30879@mail.ndmctsgh.edu.tw (Y.-H.C.); 9 Department of Family and Community Medicine, Tri-Service General Hospital, National Defense Medical Center, Taipei 11490, Taiwan

**Keywords:** artificial intelligence, type 2 diabetes, deep learning, diabetic retinopathy, fundus color photography, glycated hemoglobin

## Abstract

(1) Background: Diabetic retinopathy (DR) can cause blindness. Current guidelines on diabetic eye care recommend more frequent eye examinations for more severe DR to prevent deterioration. However, close follow-up and early intervention at earlier stages are important for the prevention of disease progression of other diabetes mellitus (DM) complications. The study was designed to investigate the association between different stages of DR in type 2 DM patients and the progression of DR; (2) Methods: A total of 2623 type 2 DM patients were included in this study. In these patients, a total of 14,409 fundus color photographs was obtained. The primary outcome was the progression of DR; (3) Results: The progression of DR was highly associated with the initial grade of DR (*p* < 0.001). Severe nonproliferative diabetic retinopathy (NPDR) was the most likely to progress to proliferative diabetic retinopathy (PDR), followed by moderate NPDR, mild NPDR, and no retinopathy. However, progression to the next stage of DR showed a different trend. We used no retinopathy as a reference. Mild NPDR showed the highest risk for progression to the next stage [hazard ratio (HR): 2.00 (95% conference interval (CI): 1.72–2.32)] relative to higher initial grades [HR (moderate NPDR): 1.82 (95% CI: 1.58–2.09) and HR (severe NPDR): 0.87 (95% CI: 0.69–1.09)]. The same trend was observed in the multivariate analysis, in which mild NPDR presented the highest risk for progression to the next stage (adjusted HR (mild NPDR): 1.95 (95% CI: 1.68–2.27), adjusted HR (moderate NPDR): 1.73 (95% CI: 1.50–1.99), and adjusted HR (severe NPDR): 0.82 (95% CI: 0.65–1.03)); (4) Conclusions: Type 2 diabetic patients with earlier-grade DR appeared to exhibit more rapid development to the next grade in our study. As these findings show, more frequent fundus color photography follow-up in earlier-grade DR patients is important to slow DR progression and awaken self-perception.

## 1. Introduction

Diabetes mellitus (DM) is an important public health issue due to diabetic complications in patients and is one of the major noncommunicable diseases globally. There will be approximately 439 million adults with diabetes worldwide in 2030. In developed countries, DM remains the biggest health issue and is predicted to increase by 69% in adults from 2010 to 2030 [1].

Diabetic retinopathy (DR), one of the most common diabetic complications, can cause blindness [2]. In Taiwan, the prevalence of DR within diabetic patients was 35% in the early 1990s, including a background DR prevalence of 30%, a preproliferative DR prevalence of 2.8%, and a proliferative diabetic retinopathy (PDR) prevalence of 2.2% [3]. The National Health Insurance database of Taiwan reported that the rate of DR prevalence increased from 6.17% to 8.91% and that of blindness increased from 0.50% to 0.62% from 2000 to 2009 [4]. Approximately one-third of diabetic patients have DR, and one-third have threatened vision globally [5].

Current studies focus on the treatment of end-stage DR. The standard treatment for PDR has been panretinal photocoagulation (PRP) since 1970. Recent studies have reported intravitreal injection as an alternative treatment [6,7]. PDR with vitreous hemorrhage and retinal detachment could be treated by vitrectomy [8]. In contrast to PDR, early-stage DR is almost asymptomatic. Patients seldom notice vision changes. Therefore, an appropriate evidence-based fundoscopy follow-up schedule is crucial to evaluate the change in the stage of DR and to limit its progression.

Deep learning models (DLMs) and artificial intelligence have made rapid progress in modern society. The DLM, having high sensitivity and specificity for identifying and grading diabetic retinopathy, is an appropriate tool for detecting diabetic retinopathy and progressive vision threats in diabetes patients. In clinical care settings, DLM could be used for following up DR stages to improve overall vision outcomes and for easing the ophthalmologist burden.

In the United Kingdom Prospective Study (UKPDS), it was shown that early management of blood sugar and hypertension in diabetic patients can delay the onset and progression of microvascular complications [9]. It is known that diabetes causes DR; however, the relationship between DR severity and the time to progression remains unclear. The study of whether the severe stage shows in more rapid deterioration than earlier stages in type 2 DM patients may guide us to determine an adequate fundoscopy follow-up period for each DR grade. Therefore, the aim of this study was to evaluate the DR progression rate in each stage via DLM follow-up.

## 2. Materials and Methods

### 2.1. Population

**Figure 1:**
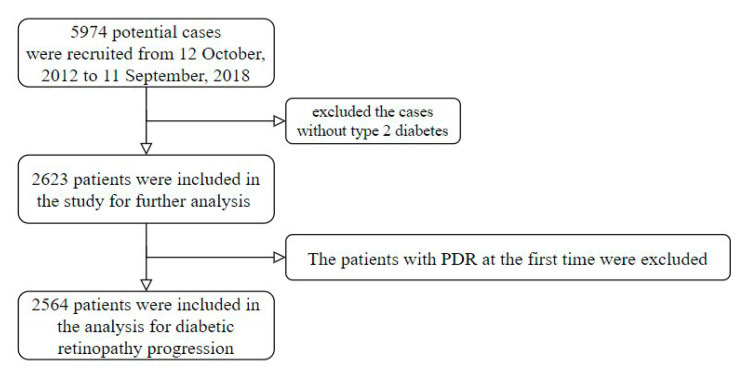
Recruitment process flow chart: 2623 patients were analyzed for baseline characteristics. Then, patients with PDR at the first time were excluded for analysis of the DR progression.

A tertiary referral medical center in Taiwan provided their research data from 12 October 2012 to 11 September 2018. The research was a retrospective study. Research ethics approval was given by the institutional review board without individual consent (IRB No. 2-105-05-073). Type 2 DM patients with more than 2 fundus color photography tests were included. The start time of follow-up was when the first fundus color photograph was obtained. There were 5974 potential cases included in this study, but we excluded patients without type 2 DM. The definition of type 2 DM was having a prescription for insulin or an oral antidiabetic and one of the following conditions: (1) at least two international classification of diseases (ICD) codes of type 2 DM (ICD-9: 250 and ICD-10: E11) at least 6 months from the start of the study; (2) at least two records of ≥126 mg/dL of blood glucose before meals (ante cibum, AC) at least 6 months from the start of the study; and (3) at least two records of ≥6.5% HbA1c at least 6 months from the start of the study. Furthermore, patients without HbA1c and fasting glucose tests within 14 days at the start time were also excluded. Finally, 2623 patients were analyzed for baseline characteristics (Figure 1). A total of 14,409 fundus color photographs was obtained from these patients. Then, patients with PDR at the first time were excluded. In total, 2564 patients were analyzed for the DR progression.

### 2.2. DLM for Grading Diabetic Retinopathy

Because it is not possible for experts to review large numbers of fundus color photography tests one by one, we used a DLM that we developed previously to grade DR [10]. The model architecture was based on a 50 layer SE-ResNeXt [11]. In addition, Kaggle [12] provided fundus color photography corresponding with DR grade for the development of the deep learning model. The public score and private score of our deep learning model in a test set involving 53,576 images were 0.837 and 0.841, respectively, which were similar to general physicians and better than optometrists [13]. The benefits of DLM are the objective evaluation to reduce subjective impact and higher efficiency, and it could be reused after training. According to the International Clinical Diabetic Retinopathy Disease Severity Scale [8], our DLM classified fundus color photography into the following 5 grades: no diabetic retinopathy; mild NPDR: microaneurysms only; moderate NPDR: any of microaneurysms, dot and blot hemorrhages, hard exudates or cotton wool spots, but less than severe NPDR; severe NPDR: intraretinal hemorrhages (≥20 in each of four quadrants), definite venous beading (in two quadrants), or intraretinal microvascular abnormalities (in one quadrant), but no signs of proliferative retinopathy; PDR: one or more of neovascularization, vitreous, or preretinal hemorrhages. Each test was conducted in both eyes. The final grade was based on the more severe eye. The definition of the end of follow-up was as follows: (1) the change in the grade of DR and (2) the end of the last fundus color photography test if there was no progression.

We collected the following laboratory records within 30 days of each fundus color photograph: total cholesterol, low-density lipoprotein (LDL), high-density lipoprotein (HDL), triglycerides, creatinine, uric acid, hemoglobin, white blood cells (WBCs), platelets, neutrophils, lymphocytes, albumin, and total bilirubin. The missing rate of the above variables in this study was less than 30%. We used multiple imputations to impute the missing values.

Other demographic characteristics and comorbidities were collected from electronic health records. The basic characteristics included sex, age, height, weight, systolic blood pressure (SBP), and diastolic blood pressure (DBP). The definition of comorbidities was based on ICD-9 and ICD-10 coding. We included the comorbidities of hypertension, ischemic heart disease, stroke, and diabetic neuropathy in our analysis as a detailed chart review.

### 2.3. Statistical Analysis and Model Performance Assessment

We presented the characteristics as the means and standard deviations (SD), numbers of patients, or percentages, where appropriate. They were compared using either analysis of variance or the chi-square test, as appropriate. We used a significance level of *p* < 0.05 throughout the analysis. The statistical analysis was carried out using the software R version 3.4.3 (Comprehensive R Archive Network, Vienna, Austria).

The primary analysis was to evaluate the effect of different grades of diabetic retinopathy progression. We used Kaplan–Meier curves to present the progression difference between participants with HbA1c and each initial grade. All variables were evaluated for their effect on diabetic retinopathy progression using a univariate Cox proportional hazard model. The multivariable Cox proportional hazard model was used to adjust the potential confounding factors, and the selection of adjusted variables was based on the significance of univariate analysis results.

## 3. Results

### 3.1. Prevalence of Different Grades of DR

We included a total of 2623 people with type 2 diabetes in this study, 1413 (56%) males and 1210 (44%) females. The variable characteristics of diabetic retinopathy at the initial test are given in Table 1. At the initial fundoscopy tests, 1046 patients (40%) had no diabetic retinopathy, 480 (18%) had mild NPDR, 756 (29%) had moderate NPDR, 282 (11%) had severe NPDR, and 59 (2%) had PDR. The initial grade of DR in our study was significantly different in terms of HbA1c (*p* < 0.001), fasting glucose (*p* < 0.001), age (*p* < 0.001), renal function (creatinine, *p* < 0.001), diabetic neuropathy (*p* = 0.001), high-density lipoprotein (HDL) (*p* = 0.039), and hemoglobin (*p* < 0.001). There were no significant associations with hypertension, blood pressure, or body mass index (BMI).

### 3.2. Effect of HbA1c on DR Progression

**Figure 2:**
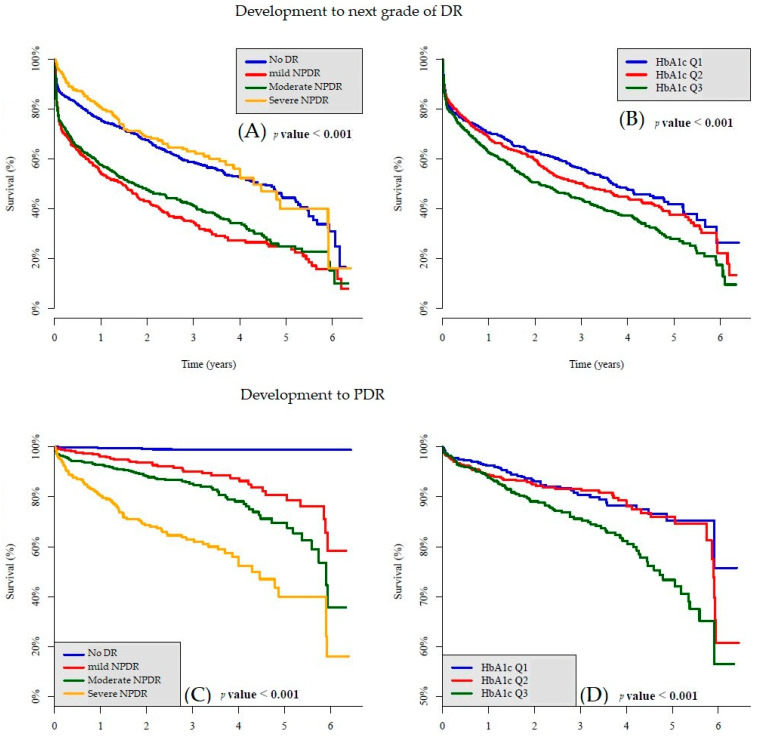
The Kaplan–Meier survival curve showed: (**A**) Development to next grade of DR comparison in each DR grade; (**B**) development to next grade of DR comparison in different tertiles of HbA1C; (**C**) development to PDR comparison in each DR grade; (**D**) development to PDR comparison in different tertiles of HbA1C. HbA1c Q1 ≤ 6.7%, HbA1c Q2 = (6.7%, 8.2%), and 8.2% < HbA1c Q3.

Considering baseline glycemic control, we divided patients into three groups equally based on HbA1c levels for subgroup analysis. Table 2 shows the characteristics of patients with different tertiles of glycemic control (by HbA1c). Subjects were divided into Q1 (HbA1c less than 6.7%), Q2 (HbA1c between 6.7% and 8.2%), and Q3 (HbA1c more than 8.2%). Age (*p* < 0.001), weight (*p* = 0.020), BMI (*p* = 0.002), systolic blood pressure (SBP) (*p* = 0.016), diastolic blood pressure (DBP) (*p* = 0.001), ischemic heart disease (*p* = 0.001), HbA1c (*p* < 0.001), fasting glucose (*p* < 0.001), triglycerides (*p* < 0.001), total cholesterol (*p* < 0.001), low-density lipoprotein (LDL) (*p* < 0.001), hemoglobin (*p* < 0.001), white blood cells (WBCs) (*p* = 0.018), and platelets (*p* < 0.001) were related to different HbA1c groups. Although SBP and DBP showed a significant correlation, the comorbidity of hypertension (*p* = 0.682) showed no relationship in the analysis. As shown in Figure 2B,D, the Kaplan–Meier survival curve showed that the higher HbA1c group was associated with faster DR progression and faster deterioration to PDR.

### 3.3. Effect of Initial Grade on DR Progression

We used a Cox proportional hazards model to identify prognostic risk factors for DR. The risk factors for progression to the next grade of DR are shown in Table 3. We estimated the difference between participants with different DR grades. All variables were evaluated for their effect on diabetic retinopathy progression by a univariate Cox proportional hazard model or a multivariable Cox proportional hazard model. Results were further adjusted for DR, gender, age, BMI, and HbA1c at baseline to assess the relative prognostic importance of each DR grade. The adjusted hazard ratios (Adj-HR) of mild NPDR (Adj-HR: 1.95; 95% CI: 1.68–2.27; *p* < 0.001), moderate NPDR (Adj-HR: 1.73; 95% CI: 1.50–1.99; *p* < 0.001), male sex (Adj-HR: 1.15; 95% CI: 1.02–1.29; *p* = 0.042), and HbA1c (Adj-HR: 1.13; 95% CI: 1.08–1.20; *p* < 0.001) were associated with the progression of diabetic retinopathy. Alanine aminotransferase (ALT) (Adj-HR: 0.87; 95% CI: 0.79–0.96; *p* = 0.003) and hemoglobin (Adj-HR: 0.89; 95% CI: 0.84–0.94; *p* = 0.001) were associated with a decreased risk of DR progression.

The risk factors for progression to PDR are shown in Table 4. Results were further adjusted for DR, gender, age, BMI, and HbA1c at baseline to assess the relative prognostic importance of each DR grade. Mild NPDR (Adj-HR: 13.53; 95% CI: 6.07–30.39; *p*-value < 0.001), moderate NPDR (Adj-HR: 23.09; 95% CI: 10.68–49.91; *p*-value < 0.001), and severe NPDR (Adj-HR: 55.24; 95% CI: 25.54–119.46; *p*-value < 0.001) were associated with progression to PDR. Age (Adj-HR: 0.73; 95% CI: 0.63–0.84; *p*-value < 0.001), DBP (Adj-HR: 1.15; 95% CI: 1.01–1.31; *p* = 0.038), and hemoglobin (Adj-HR: 0.84; 95% CI: 0.74–0.96; *p* = 0.008) were associated with a decreased risk of PDR development. Interestingly, we found no significant association between HbA1c (Adj-HR: 1.09; 95% CI: 0.97–1.22; *p* = 0.164) and progression to PDR.

As shown in the Kaplan–Meier survival curve in Figure 2A, mild NPDR and moderate NPDR (*p*-value < 0.001) were associated with faster progression to the next stage of DR than no DR and severe NPDR. Figure 2C shows that the progression of PDR occurred in a stepwise fashion, from severe NPDR to moderate NPDR to mild NPDR to no DR, because DR deterioration occurred step by step.

## 4. Discussion

Diabetic retinopathy can cause blindness, which is one of the leading causes of vision loss globally [14]. DR was reported as the fifth most common cause of preventable blindness worldwide in 2010 in a systematic analysis [15]. Our study was performed to identify the association between DR severity and prospective disease progression. Glycemic control, disease duration, and blood pressure have been reported as important risk factor for DR [16,17]. In addition to the above factors, we found that the initial severity was associated with progression of diabetic retinopathy. Severe NPDR showed easier progression to PDR, followed by moderate NPDR, mild NPDR, and no DR. However, unlike the common sense, the serious grade of DR was related to a faster deterioration. We found that mild NPDR (*p* < 0.001) and moderate NPDR (*p* < 0.001) had faster rates of progression to the next grade than no DR and severe NPDR (Figure 2A). No significant difference between the no DR group and the severe NPDR group was found.

The 2018 updated guidelines on diabetic eye care of the American Academy of Ophthalmology promote the closer monitoring of patients with more advanced disease. The recommended follow-up schedule of eye examinations is every 1–2 years for patients without DR, 6–12 months for mild NPDR, 3–6 months for moderate NPDR, 3 months for severe NPDR, 1 month for PDR, and 6–12 months for treated PDR [8]. However, the earlier grade had a faster progression rate than the end grade in our study. A close follow-up in earlier grades of DR might be helpful to prevent the deterioration of DR.

Unlike PDR, early-stage DR is almost asymptomatic and ignored. Because no direct treatment exists for early DR and the control of underlying medical conditions such as blood sugar, blood pressure, and cholesterol is the only way to slow the progression of early DR, doctors pay less attention to it. On the other hand, the standard treatment for PDR is panretinal photocoagulation (PRP) [8]. Recent studies reported the intravitreal injection of antivascular endothelial growth factor (VEGF) and ranibizumab as an alternative to PRP for PDR treatment [6,7].

In the UKPDS, glycemic control reduced or slowed diabetes complications [9], and intensive versus conventional glycemic management was associated with a 39% reduction in the risk of laser photocoagulation [18]. The result of intensive HbA1c decreasing the progression of DR was also found in our study (Figure 2B,D). However, 30–50% of patients did not meet individualized HbA1c targets at the recommended levels [19]. Diabetes self-management education (DSME) was offered by the American Diabetes Association 2015 Standards for Care as an orientation [20]. Participating in diabetes self-management education results in a decrease in HbA1c levels, which is important because glycemic control is the strongest risk factor for microvascular and macrovascular complication progression [21]. Awareness of the complications of DM has been reported as an important factor contributing to compliance with antidiabetic treatment [22,23]. Therefore, close follow-up in the early stage of DR could awake self-perception and enhance glycemic control to reduce or slow down complications of diabetes, even though no direct treatment exists for early DR.

Men had a higher risk of progression to the next grade of DR than women in our analysis, but it was not significant for the initial grade progression to end grade. The prevalence and incidence of diabetes mellitus do not differ by gender globally [24]. However, the result of diabetes complications showed gender differences. Men have a higher risk of microvascular complications than premenopausal women. However, macrovascular complications of DM are higher in women [25,26,27]. The UKPDS reported that the male sex was associated with severe retinopathy and was a risk factor for DR progression in patients with retinopathy [28,29]. These studies showed that severity of retinopathy seems to be strongly associated with male sex in the initial time of diagnosis of type 2 DM. However, the relationship between genders and diabetic retinopathy in type 2 diabetes appears to be weak when the duration of diabetes is prolonged [30]. Between men and women, differences in sex chromosomes, sex-specific gene expression and sex hormones, lifestyle, environmental influences, and nutrition may be related to the different prevalence and progression of vascular complications of diabetes [31]. Due to the lack of mechanistic studies addressing sex differences in disease pathophysiology, some remain controversial. This suggests that the progression of DR is not fully understood and dictates the need for further investigations. According to these studies, the link between male sex and progressive diabetic retinopathy weakens when the duration of diabetes is longer, strengthening the conclusion that progression to the next grade of DR is more sex specific, but sex is less significant in the progression to the end grade of DR.

Currently, the role of blood pressure in the development of diabetic retinopathy remains unclear because previous studies have provided different results about the effect of systolic blood pressure in progressive diabetic retinopathy. The previous studies showed that high blood pressure resulted in a significantly higher risk of producing DR [32,33,34]. The UKPDS 50 reported that tight blood pressure control reduced the risk of a two-step change in retinopathy grade at 12 years by 34% and emphasized the need for treatment of hypertension to reduce diabetic retinopathy [29]. Meta-analysis reported that blood pressure control reduced the relative risk of incidence of DR by 17% [35]. These studies suggested that adequate blood pressure control might be a specific approach for diabetic patients without diabetic retinopathy. Nevertheless, the current studies are not sufficient to support the association between the progression of DR and blood pressure control [35]. The results of the previous study are in accordance with the conclusion from our data showing that blood pressure is not a significant risk factor for progression to the next grade or the end grade. Yamamoto et al. reported that pulse pressure was a stronger predictor of severe DR than SBP and that adverse events were associated with patients with DBP less than 76 mmHg [36]. Elevated pulse pressure is a marker of arterial stiffness, which is an important factor in exacerbating progression of D [37]. Pulse pressure is systolic blood pressure minus diastolic blood pressure. Low DBP may indicate high pulse pressure, which is compatible with our finding that lower DBP could reduce the progression to end grade.

Some limitations of our study should be mentioned. First, this is a hospital-based retrospective study. Sampling bias and selection bias are inevitable. The results cannot be used to establish a cause–effect relationship. A community-based study should be conducted to validate these findings. Second, the stage of diabetic retinopathy was defined by DLM. The DR severity was not confirmed by an ophthalmologist due to large numbers of fundus color photography. However, our DLM demonstrated sufficient diagnostic capacity for grading DR as similar with general physicians. Third, the DM duration was not recorded in our study, although it is one of the main risk factors for DR development. However, our results showed that an early grade of DR was associated with a higher progression risk, and this implies that we may underestimate the early grade of DR. Fourth, drugs for underlying medical conditions were not evaluated in our study. Even though the results were further adjusted for DR, gender, age, BMI, and HbA1c at baseline, the additional treatment effect might have confounded the results. Finally, this analysis was performed in Taiwanese patients, and the results need to be confirmed in other populations.

## 5. Conclusions

Accounting for blood sugar control and other important characteristics, people with type 2 DM with an earlier grade of DR appear to show more rapid development to the next grade than those with a more serious grade. These findings support close fundus color photography follow-up in earlier stages of DR. It may be important to reduce DR progression and to awaken self-perception. Other risk factors for DR progression also need to be mentioned for high-risk group identification, which may help us manage the burden of diabetic complications.

## Figures and Tables

**Table 1:** The characteristics of patients with different initial grade of diabetic retinopathy.

	No DR (*n* = 1046)	Mild NPDR (*n* = 480)	Moderate NPDR (*n* = 756)	Severe NPDR (*n* = 282)	PDR (*n* = 59)	*p*-Value
Basic characteristics						
Gender						0.176
Female	506 (48.4%)	215 (44.8%)	328 (43.4%)	129 (45.7%)	32 (54.2%)	
Male	540 (51.6%)	265 (55.2%)	428 (56.6%)	153 (54.3%)	27 (45.8%)	
Age	63.85 ± 13.22	63.24 ± 12.45	59.65 ± 11.35	57.93 ± 11.81	56.66 ± 12.49	<0.001
Height (cm)	162.40 ± 8.59	162.21 ± 8.97	162.47 ± 8.73	162.56 ± 9.15	161.92 ± 9.08	0.968
Weight (kg)	67.77 ± 13.85	67.82 ± 14.02	67.81 ± 13.72	68.83 ± 15.08	68.79 ± 12.96	0.806
Body mass index	25.59 ± 4.27	25.74 ± 4.94	25.59 ± 4.27	25.91 ± 4.66	26.14 ± 3.92	0.697
SBP (mmHg)	139.02 ± 20.33	140.19 ± 21.56	141.31 ± 22.37	140.05 ± 22.93	144.76 ± 26.41	0.102
DBP (mmHg)	79.32 ± 12.15	78.20 ± 11.54	79.80 ± 12.75	80.09 ± 12.63	81.64 ± 15.08	0.084
Comorbidity						
Hypertension	376 (35.9%)	194 (40.4%)	290 (38.3%)	112 (39.7%)	25 (42.4%)	0.416
Ischemic heart disease	234 (22.4%)	119 (24.8%)	170 (22.5%)	70 (24.8%)	7 (11.9%)	0.208
Stroke	139 (13.3%)	64 (13.3%)	109 (14.4%)	30 (10.6%)	8 (13.6%)	0.641
Diabetic neuropathy	65 (6.2%)	47 (9.8%)	73 (9.6%)	19 (6.7%)	11 (18.6%)	0.001
Laboratory test						
HbA1c (%)	7.65 ± 1.87	7.96 ± 1.85	8.30 ± 2.05	8.40 ± 2.20	8.01 ± 2.05	<0.001
Fasting glucose (mg/dL)	142.11 ± 54.96	144.54 ± 52.14	154.47 ± 62.63	153.02 ± 62.83	148.37 ± 64.99	<0.001
Triglyceride (mg/dL)	150.33 ± 102.70	160.32 ± 130.04	161.75 ± 129.51	160.62 ± 138.91	158.69 ± 99.36	0.284
Total cholesterol (mg/dL)	170.36 ± 41.25	168.40 ± 46.84	171.55 ± 44.40	172.40 ± 46.86	179.27 ± 62.02	0.375
LDL (mg/dL)	99.43 ± 33.94	98.50 ± 36.38	99.94 ± 35.95	101.51 ± 36.32	107.46 ± 49.14	0.388
HDL (mg/dL)	46.90 ± 12.95	45.62 ± 12.23	45.57 ± 12.46	45.01 ± 12.68	48.32 ± 13.49	0.039
Creatinine (mg/dL)	1.31 ± 1.60	1.59 ± 1.95	1.68 ± 2.02	1.59 ± 1.72	1.93 ± 2.37	<0.001
ALT (U/L)	23.37 ± 20.28	23.62 ± 25.94	23.84 ± 39.17	22.32 ± 21.39	20.64 ± 12.46	0.877
Hemoglobin (g/dL)	13.02 ± 1.87	12.58 ± 2.00	12.36 ± 2.10	12.38 ± 2.04	11.62 ± 2.04	<0.001
White blood cell (10^3^/uL)	7.32 ± 2.77	7.54 ± 2.95	7.83 ± 6.40	7.54 ± 2.48	7.24 ± 2.45	0.138
Platelets (10^3^/uL)	212.06 ± 73.80	213.44 ± 72.56	221.54 ± 78.57	224.32 ± 66.05	220.17 ± 79.28	0.024

SBP = systolic blood pressure, DBP = diastolic blood pressure, LDL = low-density lipoprotein, HDL = high-density lipoprotein, and ALT = alanine aminotransferase.

**Table 2:** The characteristics of patients with different tertiles of baseline HbA1C.

	HbA1c ≤ 6.7% (*n* = 799)	6.7% < HbA1c ≤ 8.2% (*n* = 891)	8.2% < HbA1c (*n* = 933)	*p*-Value
Basic characteristics				
Gender				0.309
Female	358 (44.8%)	403 (45.2%)	449 (48.1%)	
Male	441 (55.2%)	488 (54.8%)	484 (51.9%)	
Age	62.08 ± 12.80	64.26 ± 11.38	59.01 ± 13.03	<0.001
Height (cm)	162.33 ± 9.00	162.56 ± 8.28	162.29 ± 9.02	0.786
Weight (kg)	66.84 ± 13.47	68.07 ± 13.78	68.71 ± 14.48	0.020
Body mass index	25.26 ± 4.07	25.67 ± 4.39	26.01 ± 4.74	0.002
SBP (mmHg)	138.39 ± 21.07	140.41 ± 20.78	141.36 ± 22.73	0.016
DBP (mmHg)	78.59 ± 12.01	78.80 ± 11.89	80.64 ± 12.97	0.001
Comorbidity				
Hypertension	311 (38.9%)	341 (38.3%)	345 (36.9%)	0.682
Ischemic heart disease	187 (23.4%)	236 (26.5%)	177 (19.0%)	0.001
Stroke	101 (12.6%)	117 (13.1%)	132 (14.1%)	0.644
Diabetic neuropathy	61 (7.6%)	63 (7.1%)	91 (9.7%)	0.090
Laboratory test				
HbA1c (%)	6.12 ± 0.43	7.41 ± 0.42	10.12 ± 1.69	<0.001
Fasting glucose (mg/dL)	124.15 ± 33.53	138.02 ± 41.28	176.33 ± 74.30	<0.001
Triglyceride (mg/dL)	134.53 ± 106.32	145.88 ± 86.40	186.12 ± 149.67	<0.001
Total cholesterol (mg/dL)	164.57 ± 39.09	166.30 ± 41.32	180.32 ± 49.60	<0.001
LDL (mg/dL)	96.19 ± 33.29	96.09 ± 32.47	106.46 ± 39.34	<0.001
HDL (mg/dL)	46.54 ± 12.86	46.45 ± 12.65	45.42 ± 12.53	0.117
Creatinine (mg/dL)	1.62 ± 2.08	1.47 ± 1.70	1.45 ± 1.74	0.107
ALT (U/L)	22.53 ± 20.98	23.07 ± 18.91	24.39 ± 38.45	0.355
Hemoglobin (g/dL)	12.42 ± 2.06	12.64 ± 1.96	12.86 ± 2.00	<0.001
White blood cell (10^3^/uL)	7.26 ± 4.73	7.47 ± 4.66	7.82 ± 2.93	0.018
Platelets (10^3^/uL)	203.70 ± 70.38	212.64 ± 69.60	231.26 ± 79.68	<0.001

SBP = systolic blood pressure, DBP = diastolic blood pressure, LDL = low-density lipoprotein, HDL = high-density lipoprotein, and ALT = alanine aminotransferase.

**Table 3:** The risk of progression to next grade at 6 year visits by Cox proportional hazard model.

	Crude-HR (95% CI)	*p*-Value	Adjusted-HR (95% CI)	*p*-Value
Initial grade		<0.001		<0.001
No DR	1.00		1.00	
Mild NPDR	2.00 (1.72–2.32)	<0.001	1.95 (1.68–2.27)	<0.001
Moderate NPDR	1.82 (1.58–2.09)	<0.001	1.73 (1.50–1.99)	<0.001
Severe NPDR	0.87 (0.69–1.09)	0.223	0.82 (0.65–1.03)	0.082
Basic characteristics				
Gender		0.019		0.042
Female	1.00		1.00	
Male	1.15 (1.02–1.29)	0.019	1.13 (1.00–1.27)	0.042
Age	0.95 (0.90–1.01)	0.076	0.97 (0.91–1.03)	0.275
Height	1.06 (1.00–1.12)	0.057	1.05 (0.99–1.12)	0.095
Weight	1.02 (0.96–1.08)	0.474	1.08 (0.97–1.22)	0.169
Body mass index	1.00 (0.94–1.06)	0.876	0.99 (0.94–1.05)	0.802
SBP	1.03 (0.97–1.09)	0.309	1.02 (0.96–1.08)	0.542
DBP	1.04 (0.98–1.10)	0.201	1.03 (0.97–1.10)	0.287
Comorbidity				
Hypertension	1.11 (0.99–1.25)	0.087	1.08 (0.96–1.22)	0.197
Ischemic heart disease	1.06 (0.92–1.21)	0.417	1.07 (0.94–1.23)	0.319
Stroke	1.15 (0.97–1.35)	0.104	1.11 (0.94–1.31)	0.203
Diabetic neuropathy	1.23 (1.01–1.50)	0.043	1.11 (0.91–1.36)	0.311
Laboratory test				
HbA1c	1.13 (1.08–1.20)	<0.001	1.11 (1.05–1.17)	<0.001
Fasting glucose	1.04 (0.98–1.10)	0.173	0.97 (0.91–1.03)	0.302
Triglyceride	1.01 (0.95–1.06)	0.784	0.97 (0.91–1.03)	0.343
Total cholesterol	1.00 (0.94–1.06)	0.966	0.98 (0.92–1.04)	0.537
LDL cholesterol	1.01 (0.95–1.08)	0.682	0.99 (0.93–1.06)	0.821
HDL cholesterol	0.95 (0.90–1.01)	0.101	0.98 (0.92–1.04)	0.458
Creatinine	1.05 (1.00–1.11)	0.061	1.02 (0.96–1.07)	0.568
ALT	0.87 (0.79–0.96)	0.005	0.86 (0.79–0.95)	0.003
Hemoglobin	0.89 (0.84–0.94)	<0.001	0.91 (0.86–0.96)	0.001
White blood cell	1.06 (1.01–1.11)	0.022	1.04 (0.99–1.09)	0.159
Platelets	1.06 (1.00–1.12)	0.037	1.04 (0.98–1.10)	0.199

All result of Adjusted HR were adjusted by initial DR, gender, age, BMI, HbA1c; the continuous variables are standardized by mean and standard deviation; therefore, the units of each continuous variable were 1 standard deviation; HR = hazard ratios, CI = confidence interval, SBP = systolic blood pressure, DBP = diastolic blood pressure, LDL =low-density lipoprotein, HDL = high-density lipoprotein, and ALT = alanine aminotransferase.

**Table 4:** The risk of progression to PDR at 6 year visits by Cox proportional hazard model.

	Crude-HR (95% CI)	*p*-Value	Adjusted-HR (95% CI)	*p*-Value
Initial grade		<0.001		<0.001
No DR	1.00		1.00	
Mild NPDR	13.83 (6.19–30.93)	<0.001	13.58 (6.07–30.39)	<0.001
Moderate NPDR	25.06 (11.61–54.08)	<0.001	23.09 (10.68–49.91)	<0.001
Severe NPDR	62.29 (28.87–134.40)	<0.001	55.24 (25.54–119.46)	<0.001
Basic characteristics				
Gender		0.422		0.750
Female	1.00		1.00	
Male	1.11 (0.86–1.44)	0.422	0.96 (0.74–1.25)	0.750
Age	0.67 (0.60–0.76)	<0.001	0.73 (0.63–0.84)	<0.001
Height	1.04 (0.92–1.19)	0.522	0.97 (0.84–1.12)	0.671
Weight	0.98 (0.86–1.12)	0.789	0.93 (0.71–1.20)	0.569
Body mass index	0.95 (0.83–1.09)	0.470	0.91 (0.79–1.03)	0.146
SBP	1.10 (0.97–1.25)	0.137	1.11 (0.98–1.25)	0.111
DBP	1.23 (1.08–1.40)	0.001	1.15 (1.01–1.31)	0.038
Comorbidity				
Hypertension	1.18 (0.91–1.54)	0.210	1.11 (0.86–1.45)	0.421
Ischemic heart disease	1.13 (0.84–1.52)	0.414	1.11 (0.82–1.50)	0.498
Stroke	1.61 (1.16–2.25)	0.005	1.72 (1.23–2.40)	0.001
Diabetic neuropathy	1.45 (0.95–2.21)	0.087	1.15 (0.75–1.78)	0.519
Laboratory test				
HbA1c	1.30 (1.16–1.46)	<0.001	1.09 (0.97–1.22)	0.164
Fasting glucose	1.13 (1.01–1.26)	0.032	0.93 (0.82–1.06)	0.263
Triglyceride	1.05 (0.94–1.17)	0.397	1.01 (0.91–1.12)	0.878
Total cholesterol	1.01 (0.88–1.16)	0.916	0.93 (0.81–1.07)	0.305
LDL	1.00 (0.87–1.14)	0.963	0.89 (0.78–1.03)	0.120
HDL	0.84 (0.74–0.97)	0.017	0.88 (0.76–1.01)	0.069
Creatinine	1.17 (1.05–1.29)	0.003	1.11 (0.99–1.23)	0.062
ALT	0.89 (0.72–1.10)	0.285	0.92 (0.76–1.12)	0.425
Hemoglobin	0.74 (0.65–0.84)	<0.001	0.84 (0.74–0.96)	0.008
White blood cell	1.08 (0.97–1.21)	0.148	1.03 (0.89–1.19)	0.686
Platelets	1.14 (1.01–1.28)	0.033	1.05 (0.92–1.20)	0.493

All result of adjusted-HR were adjusted by initial DR, gender, age, BMI, and HbA1c; the continuous variables are standardized by mean and standard deviation; therefore, the units of each continuous variable were 1 standard deviation; HR = hazard ratios, CI = confidence interval, SBP = systolic blood pressure, DBP = diastolic blood pressure, LDL = low-density lipoprotein, HDL = high-density lipoprotein, and ALT = alanine aminotransferase.

## Data Availability

Data available in a publicly accessible repository that does not issue DOIs. The data presented in this study are openly available in kaggle; reference number [12].
